# Molecular-Scale Regulation of Cement Hydration and Microstructure via Synergistic Aluminum Sulfate–Amide Interactions

**DOI:** 10.3390/ma19163538

**Published:** 2026-08-20

**Authors:** Chuanjiu Zhang, Jie Chen, Hu Chen, Peng Li, Kaiwen Shi, Fei Gao, Xuanliang Li, Qiangqiang Hu, Meng Li

**Affiliations:** 1 School of Resources and Safety Engineering, Chongqing University, Chongqing 400044, China; 2 China Energy Shendong Coal Group Co., Ltd., Ordos 017200, China; 3 CCTEG Wuhan Engineering Company, Wuhan 430064, China; 4 College of Energy and Mining Engineering, Xi’an University of Science and Technology, Xi’an 710054, China

**Keywords:** alkali-free accelerator, aluminum sulfate, amide synergy, cement hydration, density functional theory

## Abstract

High-performance alkali-free accelerators require a mechanistic understanding of interactions between inorganic accelerants and organic modifiers. Although aluminum sulfate (AS) promotes rapid ettringite (AFt) formation, uncontrolled crystallization leads to coarse microstructures and instability from AFt-to-AFm conversion. Here, a molecular-scale synergistic mechanism is identified in which an amide regulates AS-driven hydration. The amide controls nucleation and growth of AFt and C–S–H via chemisorption on C_3_A/C_3_S and complexation with Ca^2+^. Within the tested mixing proportions, the high-aluminum-sulfate and moderate-amide combination achieves the highest early strength (17.05 MPa at 1 day) via constructing an interlocked AFt/C–S–H skeleton, whereas excessive amide suppresses crystallization and low AS accelerates AFt-to-AFm conversion, reducing long-term performance. In-situ XRD, thermal analysis, and microscopy confirm a denser, more stable microstructure (27.37 MPa at 10 days) with minimal 28-day strength loss (17.38 MPa). Density functional theory shows an adsorption hierarchy of amide > AS species > H_2_O, explaining its dominant surface-modifying role. This study provides a framework for designing cement accelerators with balanced early strength and durability.

## 1. Introduction

The exigencies of modern underground engineering and high-speed precast construction place stringent demands on cementitious materials, particularly for rapid setting and high early-age strength development [1,2,3]. Chemical accelerators are indispensable in meeting these requirements. However, traditional chloride-based accelerators induce corrosion of steel reinforcement, and conventional alkaline accelerators pose risks of alkali-silica reaction and long-term durability issues. Consequently, alkali-free liquid accelerators (AFLAs) based on aluminum sulfate (AS) have gained prominence due to their favorable durability profile [4,5,6,7].

Extensive prior work has confirmed the efficacy of AS in promoting early hydration. Xu et al. demonstrated that AS-based systems can reduce cement initial setting time to <5 min, while Rasid et al. reported 1-day strength improvements of up to 200% compared to plain cement [8,9]. However, these studies also consistently highlight inherent limitations of AS-only systems. Uncontrolled AFt crystallization yields heterogeneous, porous microstructures with poor interparticle interlocking [10,11], and more critically, the metastable AFt phase undergoes conversion to monosulfoaluminate (AFm) over time. This phase transformation is accompanied by ~30% solid volume reduction, leading to significant strength retrogression—reported to reach 30–40% at 28 d in several AS-dominated formulations [12,13]. To mitigate these drawbacks, composite accelerators incorporating organic modifiers have become a key research focus. Among these, amide compounds have garnered particular interest due to their polar -CONH_2_ groups, which enable adsorption onto cement mineral surfaces and complexation with Ca^2+^ in pore solution to modulate hydration kinetics [14,15]. Wanget al. and Wang et al. observed that amide addition can refine AFt crystal size and promote C-S-H gel uniformity, yet these regulatory effects were found to be highly dose-dependent [16,17].

Nevertheless, the current understanding of the synergistic mechanism between AS and amide compounds remains largely phenomenological rather than mechanistic [18,19]. A critical and unresolved question is how the specific dosage ratio of AS to the amide compound precisely governs the hydration pathway, steering the interplay between the aluminate reaction (AFt formation) and the silicate reaction (C–S–H gel formation), and ultimately dictating the evolution of the microstructure and the final mechanical properties [20,21]. This lack of a predictive, quantitative model grounded in chemical principles hinders the rational design of next-generation, high-performance composite accelerators [22,23].

In this work, we hypothesize that the amide compound functions as a molecular-scale regulator that fine-tunes the rapid crystallization of AFt driven by AS, while simultaneously promoting a more uniform and dense precipitation of C-S-H gel. This study aims to systematically decode this synergy. We employ a combined experimental approach, correlating macroscopic properties with microstructural evolution for cement pastes incorporating varying dosages of AS and an amide compound. The ultimate aim is to construct a coherent reaction model that clearly delineates the respective and interactive roles of ionic precipitation kinetics (governed by AS) and adsorption-modulated nucleation (governed by the amide). The findings of this study are expected to provide fundamental insights for the chemical engineering of cement hydration processes and the targeted design of advanced AFLAs.

## 2. Experimental Section

### 2.1. Raw Materials

**Figure 1:**
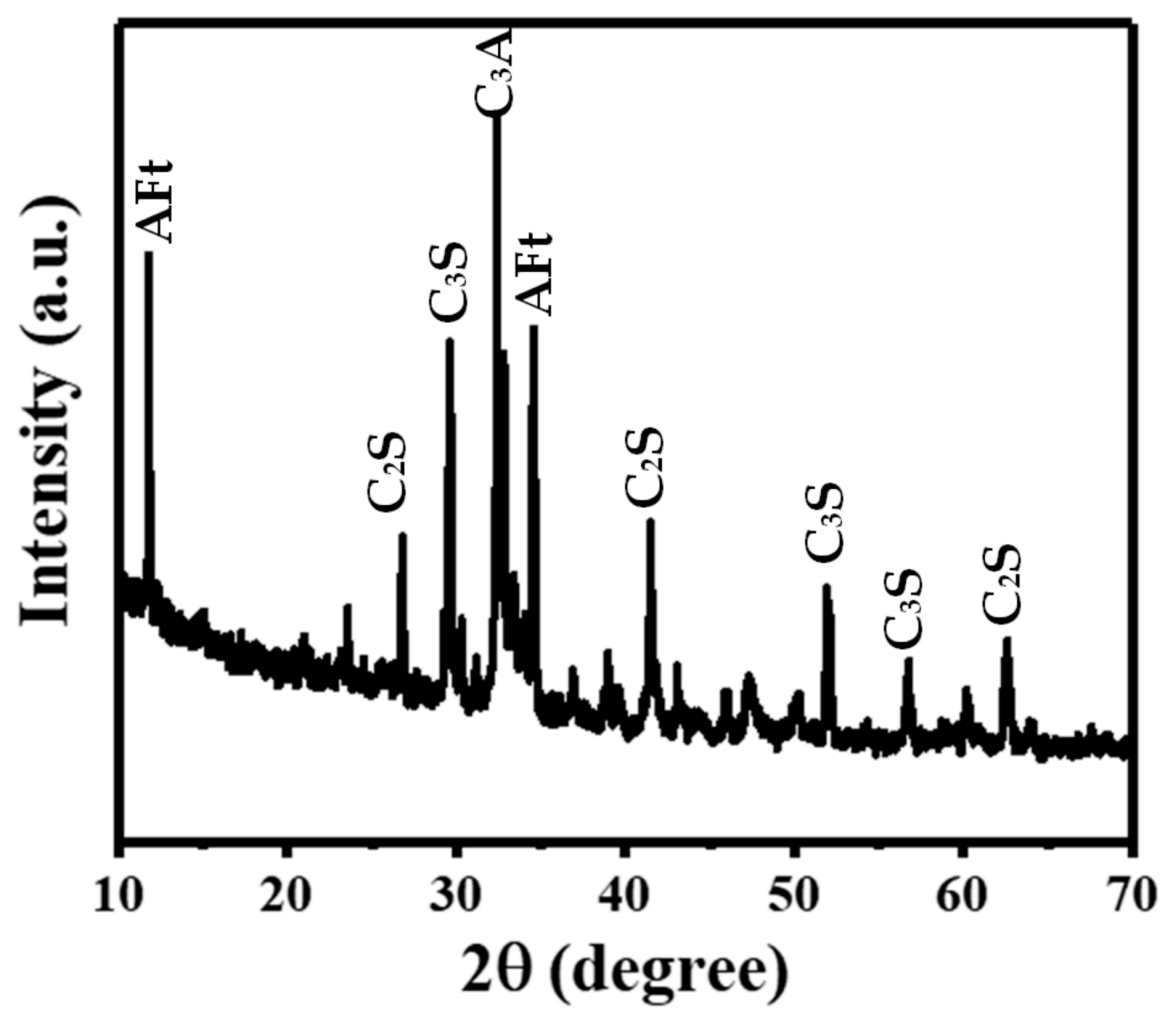
X-ray diffraction pattern of OPC.

The main raw materials used in this study are detailed below: ordinary portland cement (OPC) of grade P.O 42.5 was used. Its physical properties and chemical composition comply with the Chinese National Standard GB 175-2007 Common Portland Cement [24]. As shown in Figure 1, the XRD pattern exhibits several characteristic diffraction peaks corresponding to the major clinker phases. The strongest peaks at approximately 32.8°, 53.5° and 57.1° are assigned to alite (C_3_S, PDF 01-070-0285), while the peaks around 29.5°, 41.8° and 62.3° are attributed to belite (C_2_S, PDF 00-033-0302). Additional peaks at approximately 33.2° correspond to tricalcium aluminate (C_3_A, PDF 00-038-1429), the sharp peak at approximately 9.1° corresponds to ettringite (AFt, PDF 00-041-1451). These diffraction features confirm the typical crystalline phase assemblage of P.O 42.5 cement clinker. Calcium aluminate (industrial grade) was used to adjust the setting time and enhance early-age strength development. Aluminum sulfate (analytical grade) was employed as an inorganic salt activator to participate in the reaction and influence the microstructure of the hardened matrix. An amide-based compound (DMAC, C_4_H_9_NO) was utilized as an organic polymer additive, primarily to modify the rheology of the fresh mixture or act as a water-retaining/retarding agent. Clean tap water from the laboratory supply.

### 2.2. Experimental Mix Proportions

The backfill aggregate selected for this test was cement, with the total dosage of the additive set at 7% (by mass of cement). The additive was composed of aluminum sulfate, high alumina salt, an amidation reaction product, and water. Four distinct experimental mix proportions were designed by varying the dosages of aluminum sulfate and the amidation reaction product within this 7% total additive. The detailed mix formulations are presented in Table 1.

To ensure a constant effective w/c ratio of 0.35 across all mixtures, the total water content (sum of external mixing water and water incorporated within the liquid additive) was fixed at 140 g per 400 g of cement. Variations in additive solid content were compensated by inversely adjusting the external mixing water.

### 2.3. Test Methods

For the compressive strength tests, the cement paste mixtures were cast into standard molds with dimensions of 40 mm × 40 mm × 40 mm. After casting, the specimens were cured in a standard moist cabinet at a temperature of 20 ± 2 °C and a relative humidity of strictly ≥95% until the specified testing ages of 1, 10, and 28 days. For each mixture and testing age, a minimum of 3 replicate specimens were tested. The final compressive strength values are reported as the average of these replicates, and the standard deviations were calculated to ensure statistical reliability.

### 2.4. Characterizations

XRD patterns were collected on a Bruker D8 Advance diffractometer (Bruker AXS GmbH, Karlsruhe, Germany) equipped with Cu Kα radiation (λ = 1.5406 Å). The measurements were performed at 40 kV and 40 mA, scanning over a 2θ range of 5–70° with a scan rate of 2°/min.; SEM imaging was performed on a ZEISS GeminiSEM 300 microscope (Carl Zeiss Microscopy GmbH, Jena, Germany) operated at 15 kV, after gold sputter-coating of fractured and ethanol-stopped hydrated samples.; Thermogravimetric analysis was conducted on a NETZSCH STA 449 F3 simultaneous thermal analyzer (NETZSCH-Gerätebau GmbH, Selb, Germany) under N_2_ atmosphere from 30 to 900 °C at a heating rate of 10 °C/min.; Zeta potential measurements were carried out on a Malvern Zetasizer Nano ZS (Malvern Instruments Ltd., Malvern, Worcestershire, UK) using freshly prepared cement suspensions (solid-to-liquid ratio of 1:10) diluted to avoid multiple scattering effects.; and compressive strength tests were performed on a servo-hydraulic testing machine at a loading rate of 2.4 kN/s following GB/T 50081-2019 [25]. DFT calculations are also now explicitly noted to use the Cambridge Serial Total Energy Package (CASTEP) module (version 24.1, embedded in BIOVIA Materials Studio 2024) with ultrasoft pseudopotentials and the GGA-PBE exchange-correlation functional, with a plane-wave cutoff energy of 450 eV and k-point spacing of 0.04 Å^−1^.

## 3. Results and Discussion

### 3.1. Mechanism of the Early-Strength Agent

**Figure 2:**
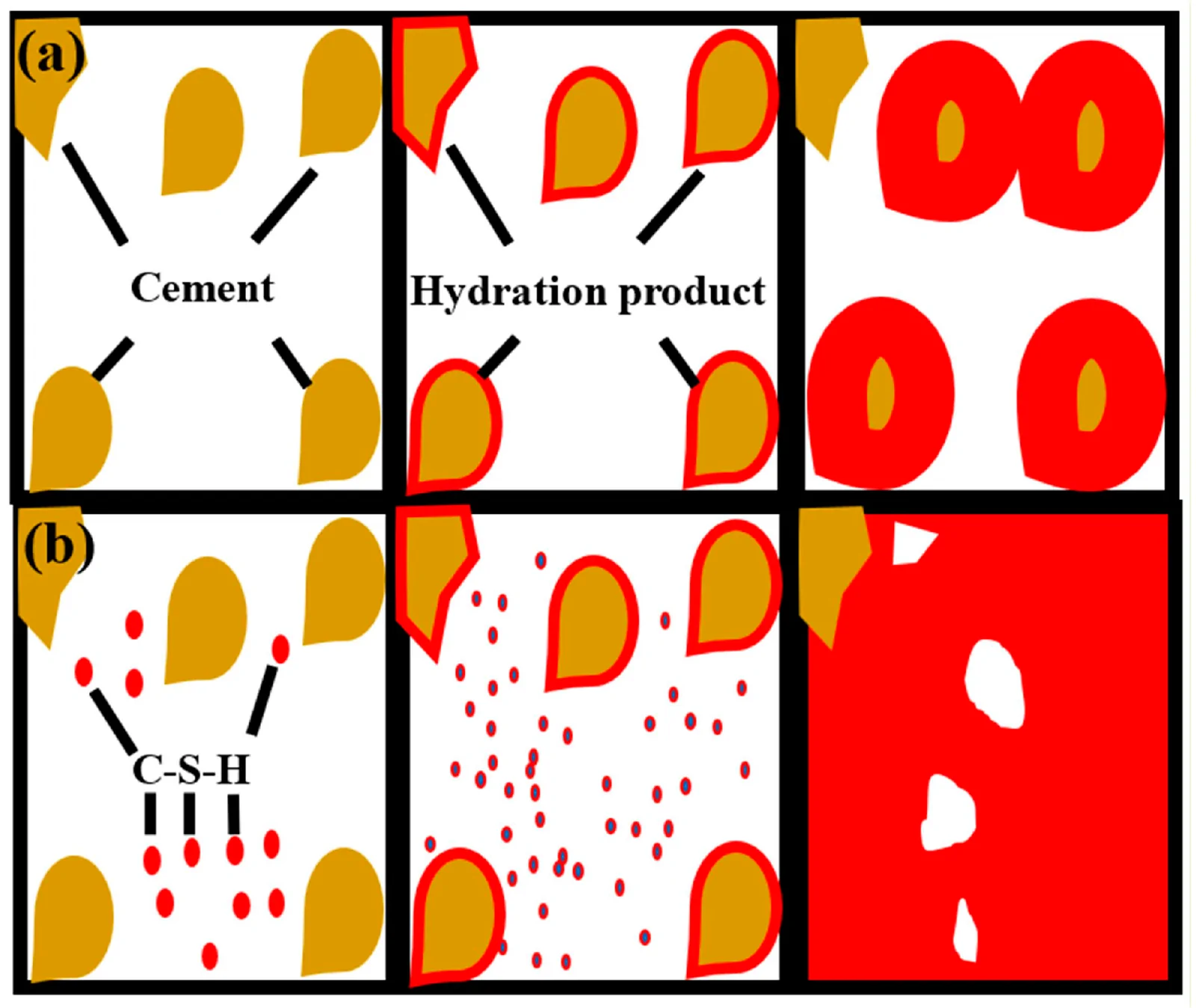
Schematic diagram of the harden mechanism (**a**) Blank cement past (**b**) cement paste containing accelerator.

As shown in Figure 2a, during the initial stage of hydration, the energy barrier for homogeneous nucleation in the bulk pore solution is significantly higher than that required for heterogeneous nucleation on existing surfaces. Consequently, hydration products preferentially precipitate on the surfaces of cement clinker minerals. By incorporating an early strength agent, finely divided particles and reactive species are introduced into the system, acting as exogenous nucleation sites (Figure 2b). Cement hydration products such as ettringite and C-S-H gel can then grow on these pre-existing surfaces, which drastically lowers the activation energy for nucleation by reducing the interfacial energy demands. This process is governed by the principles of heterogeneous nucleation, where the presence of a substrate decreases the thermodynamic barrier to crystal formation. Because hydration can occur simultaneously at multiple locations across these dispersed sites, the early-age microstructure becomes significantly denser. A greater quantity of effective nucleation sites leads to a higher degree of hydration within the first 24 h, enhancing the interlocking of nascent phases and accelerating the gain of mechanical strength.

### 3.2. Setting Time and Compressive Strength

**Figure 3:**
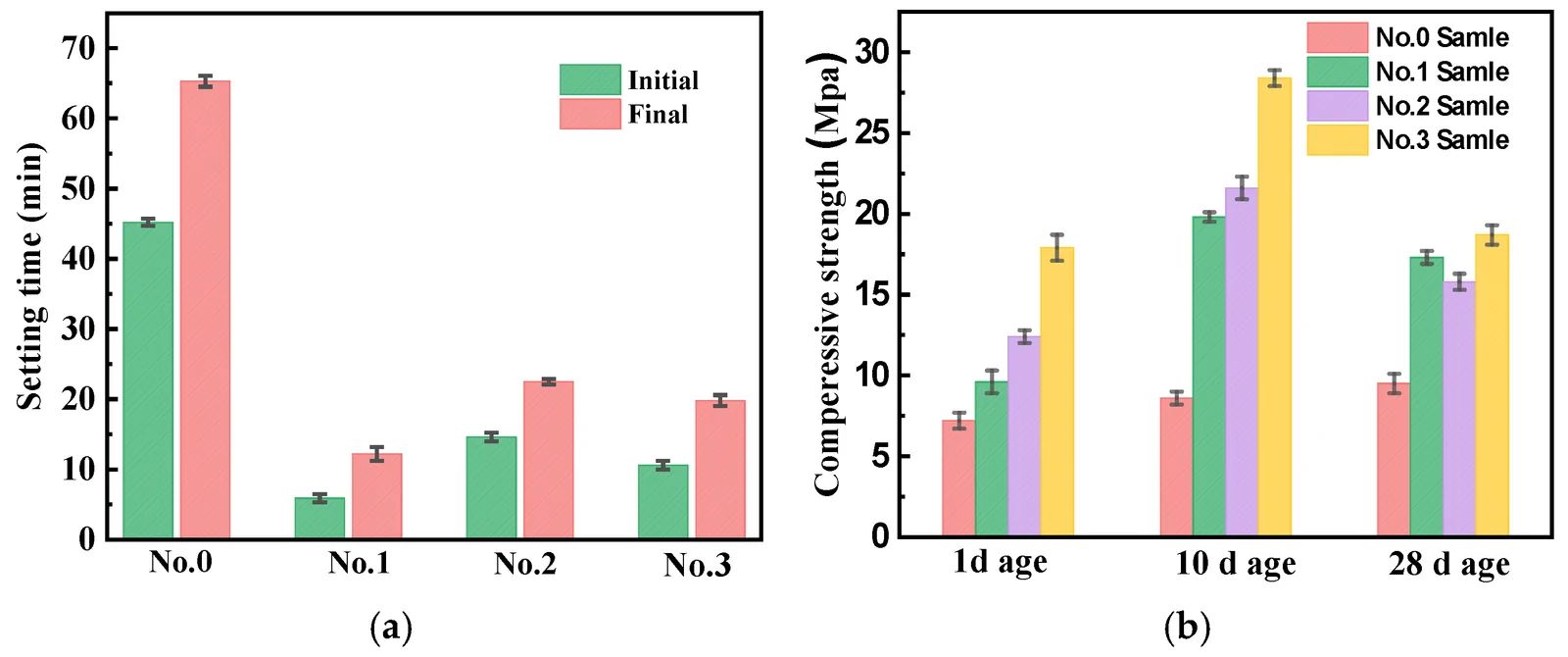
(**a**) Effect of aluminum sulfate and amide compound on setting time of cement paste. (**b**) Effect of aluminum sulfate and amide compound on compressive strength of cement paste.

The setting behavior of cement paste is a direct macroscopic manifestation of early-stage hydration kinetics. As shown in Figure 3a, the control sample (No. 0) exhibited initial and final setting times of 45 and 65 min, respectively. The incorporation of the aluminum sulfate-amide (AS-Am) composite accelerator significantly shortened these times, confirming its role as an effective setting promoter. Crucially, the impact was highly dependent on the specific dosage ratio of the two components.

Comparing samples No. 1 and No. 3 (constant high AS, varying amide), a reduction in the amide dosage led to a progressive prolongation of both the initial and final setting times. This phenomenon can be attributed to the adsorption and complexation mechanism of the amide compound during the cement hydration process. Functional groups within the amide compound molecule, such as the amide group (-CONH_2_), exhibit a strong affinity for adsorption onto the surfaces of cement mineral phases (e.g., C_3_A, C_3_S). Concurrently, these molecules can chelate calcium ions (Ca^2+^) present in the pore solution. This adsorption disrupts the diffuse double-layer structure on the cement particle surfaces, promoting particle flocculation. Moreover, the formed complexes provide additional nucleation sites for the precipitation of key hydration products, such as ettringite and C-S-H gel, thereby significantly enhancing the kinetics of the hydration reactions [26]. Consequently, a decrease in the amide compound dosage attenuates this catalytic adsorption-nucleation effect, leading to a corresponding prolongation of the setting times.

On the other hand, when the dosage of the amide compound was maintained constant (Samples No. 1 and No. 2), a decrease in the aluminum sulfate content similarly led to delayed setting. This is primarily due to the critical role of aluminum sulfate in regulating the early-stage formation of AFt. Upon dissolution, aluminum sulfate rapidly releases a substantial quantity of aluminum ions (Al^3+^) and sulfate ions (SO_4_^2−^) into the system. These Al^3+^ ions readily participate in rapid reactions with Ca^2+^, hydroxide (OH^−^), and SO_4_^2−^ dissolved from the cement clinker during the initial hydration period, leading to the precipitation of needle-like ettringite crystals [27,28]. The rapid formation and subsequent intergrowth of a substantial amount of these crystals construct the primary skeletal framework of the cement paste at early ages. This microstructural development is responsible for the loss of workability and the onset of strength gain. The lowered ionic concentration directly reduces the supersaturation and nucleation rate of AFt, retarding framework formation [29,30,31].

The compressive strength results (Table 2 and Figure 3b) clearly demonstrate the significant influence of aluminum sulfate and amide compound dosages on the strength development of cement paste.

A detailed analysis shows that the 1-day strength of all modified samples (ranging from 9.37 ± 0.71 MPa to 17.05 ± 0.81 MPa) was significantly higher than that of the control (6.41 ± 0.53MPa), indicating a considerable acceleration of early-age hydration by the additives. Notably, the dosage of the amide compound plays a dominant role in early strength development. Comparing sample No. 1 and No. 3 sample, which share the same aluminum sulfate dosage (3.5%), a reduction in the amide content led to a remarkable increase in 1-day strength from 9.37 MPa to 17.05 MPa. This counterintuitive phenomenon suggests that excessive amide inhibits early microstructural development. The mechanism involves strong adsorption and steric hindrance from high amide dosage, which disrupts the oriented growth and interlocking of AFt and C–S–H crystals, weakening the early skeleton. In contrast, moderate amide dosage promotes nucleation while minimizing growth interference.

By the 10-day curing age, the strength of all samples continued to increase. At this stage, the role of aluminum sulfate in providing the strength-bearing skeleton becomes more critical. It was observed that the 10-day strength of sample No. 1 (19.81 MPa ± 0.36 MPa) was slightly lower than that of No. 2 sample (20.87 MPa ± 0.75 MPa). This can be attributed to the excessively high ion concentration caused by a high aluminum sulfate dosage, potentially leading to rapid ettringite (AFt) crystallization, which induces localized stress concentration or microstructural defects. A moderate aluminum sulfate dosage (e.g., 2.1%), however, favors the formation of a more homogeneous and denser interwoven AFt/C-S-H microstructure. Furthermore, in sample No. 3 with a reduced amide dosage, the diminished inhibitory effect of the amide compound on initial hydration allows for a better match between the AFt formation rate promoted by aluminum sulfate and the hydration rate of C_3_S. This synergy achieves optimal microstructural densification at 10 days, yielding the highest compressive strength (27.37 MPa ± 0.52 MPa) among the three modified mixtures.

A key phenomenon observed at the 28-day age was the occurrence of varying degrees of strength retrogression in all additive-containing samples. This behavior is attributed to AFt-to-AFm conversion. Sample No. 3 exhibited the highest absolute 28 d compressive strength among all accelerator modified mixtures, despite undergoing the largest relative strength loss from 10 to 28 d, indicating that the dense AFt/C-S-H intergrowth in Sample No. 3 contributes to the highest residual strength, although the relative strength retrogression remains most pronounced due to extensive AFt to AFm conversion. The dense AFt/C–S–H intergrowth reduces local permeability and stabilizes pore solution chemistry, delaying AFt conversion. Samples No. 1 and No. 2, with more porous or heterogeneous microstructures, experienced greater strength loss.

To quantitatively evaluate the long-term strength degradation induced by AFt-to-AFm transformation, the relative strength loss rate from 10 d to 28 d was calculated for each modified group. Sample No. 1 undergoes a 16.7% strength drop from 19.81 MPa to 16.50 MPa, Sample No. 2 presents a 26.9% loss from 20.87 MPa to 15.26 MPa, and Sample No. 3 suffers a 36.5% reduction from its peak 10 d strength of 27.37 MPa down to 17.38 MPa. The target application of this AS-amide composite alkali-free accelerator is underground shotcrete for temporary early support in tunnels and mine roadways, whose core performance demands high strength within 24–72 h for instant surrounding rock stabilization, with 28 d strength serving as secondary long-term guarantee. The 28 d compressive strength of Sample No. 3 reaches 17.38 MPa, surpassing the 15 MPa minimum 28 d strength threshold specified by ASTM C1140/C1140M for structural shotcrete; meanwhile, its 1 d strength of 17.05 MPa is nearly three times that of the blank reference group, fully satisfying the core requirement of ultra-fast early hardening. Even though Sample No. 3 delivers the highest absolute residual strength at 28 d among all accelerator-containing mixtures, the 36.5% relative strength retrogression still reveals insufficient long-term stability. This limitation is attributed to inevitable AFt-to-AFm phase transformation under prolonged hydration, and relevant optimization strategies will be prioritized in follow-up research to suppress this phase conversion and cut down long-term strength loss.

### 3.3. XRD Analysis of Hydration Products of Cement Paste

**Figure 4:**
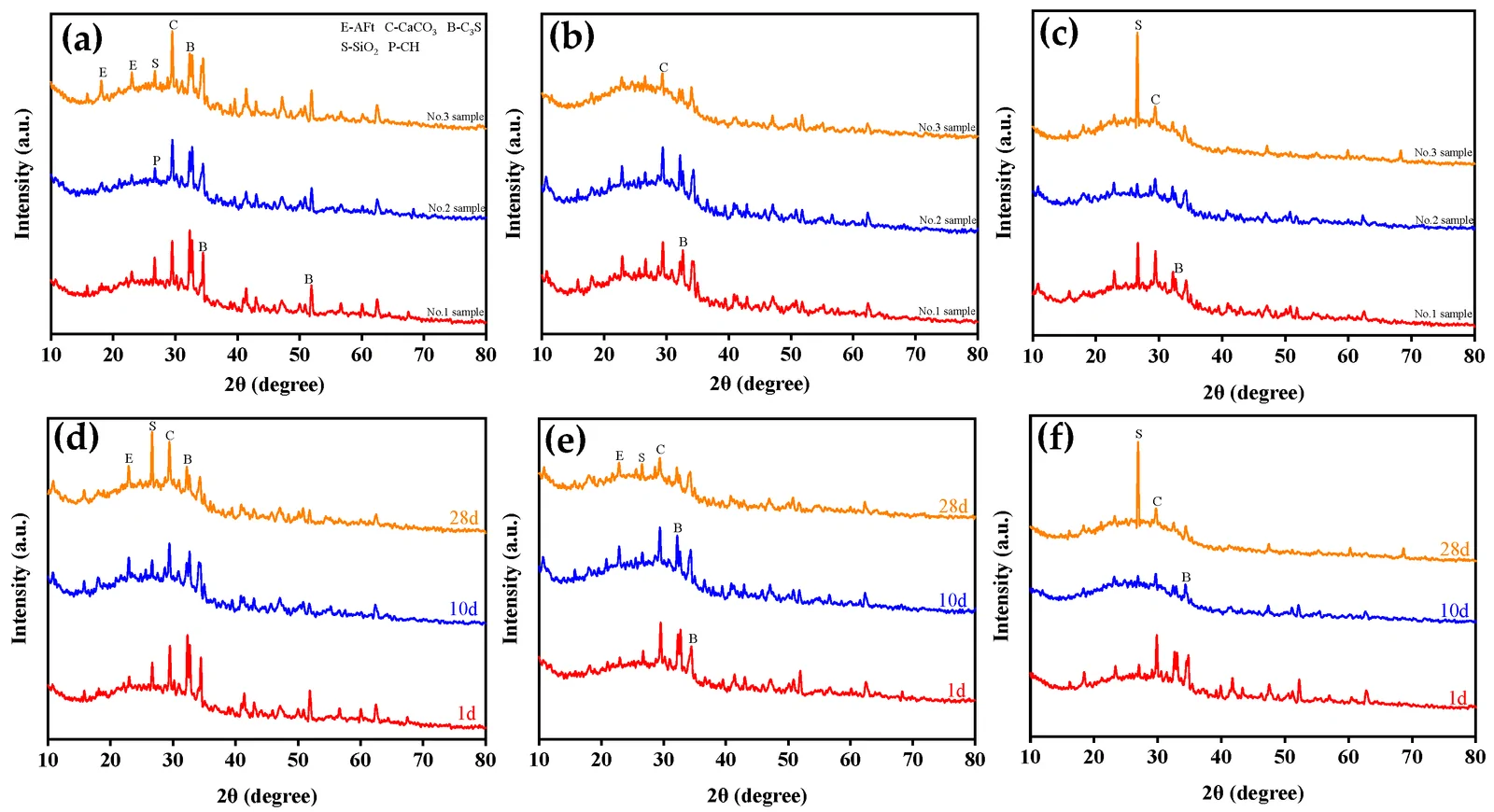
(**a**) hydration for 1 d of different samples, (**b**) hydration for 10 d of different samples, (**c**) hydration for 28 d of different samples, (**d**) hydration for the No. 1 sample in each age, (**e**) Hydration for the No. 2 sample in each age, (**f**) Hydration for the No. 3 sample in each age.

To gain deeper insights into the mechanistic effects of aluminum sulfate and the amide compound on the cement hydration process at the micro-scale, X-ray diffraction (XRD) analysis was performed on samples with different formulations at various curing ages, as shown in Figure 4. Specifically, Ettringite (AFt): PDF#00-041-1451, Portlandite (CH): PDF#00-044-1481, Calcite (CaCO_3_): PDF#00-047-1743, Quartz (SiO_2_): PDF#00-046-1045, C_3_S (Alite): PDF#01-070-0285. At 1 day, the positions and profiles of the primary diffraction peaks were largely consistent across all samples. The characteristic peaks of AFt, portlandite (CH), and unhydrated C_3_S/C_2_S were observed, confirming that additives primarily modulate crystallization kinetics rather than induce new crystalline phases [32,33].

After 10 days, significant differences in phase evolution emerged. Sample No. 3 showed the most pronounced attenuation of C_3_S diffraction intensity, indicating accelerated silicate reaction kinetics. This enhancement originates from the amide’s dual regulatory function: moderate adsorption reduces surface blockage while maintaining sufficient nucleation sites, facilitating both C_3_S dissolution and C–S–H precipitation. Concurrently, the relatively stable AFt diffraction patterns in this sample are consistent with partial preservation of the primary crystalline framework.

By 28 days, the divergence in phase composition became more pronounced. Sample No. 3 exhibited near-complete consumption of C_3_S phases, with the quartz (SiO_2_) impurity peak becoming proportionally prominent. While not a quantitative measure, this relative change in peak intensity is consistent with a reduction in overall crystalline phases, which often accompanies the extensive formation of amorphous C-S-H gel. In contrast, the characteristic peaks in Sample No. 2 were universally weaker than those in Sample No. 1. This is primarily due to the instability of hydration products resulting from insufficient aluminum sulfate. The lack of adequate SO_4_^2−^ and Al^3+^ ions compromises the long-term stability of AFt, leading to its partial conversion to AFm [34,35,36]. This phase transformation is accompanied by a deterioration of the microstructure and a decrease in crystallinity, manifesting as peak weakening, and is directly correlated with the significant strength retrogression observed in this sample.

Furthermore, a longitudinal comparison of phase evolution reveals distinct hydration pathways: In sample No. 1 the continuous attenuation of the C_3_S peak indicates ongoing silicate hydration, while the apparent strengthening of the SiO_2_ peak suggests a relative decrease in other diffracting crystalline phases, aligning with the expected accumulation of amorphous C-S-H gel. Sample No. 2 exhibits a systematic weakening of all major peaks over time. This suggests poor crystallinity and long-term instability of the hydration products (especially the AFt/AFm system), rather than simply high hydration degree, consistent with its severe strength retrogression and indicating an inability to form a stable, well-ordered crystalline matrix. Conversely, the evolution of Sample No. 3 is the most optimal. The near-complete disappearance of the C_3_S phase, alongside the altered relative intensities in the 28 d diffraction pattern, supports the premise of a higher hydration degree and the most thorough conversion into hydration products (predominantly C-S-H gel). This results in the densest microstructure, offering a direct explanation for its exceptional long-term mechanical performance.

### 3.4. SEM Analysis of Hydration Products of Cement Paste

**Figure 5:**
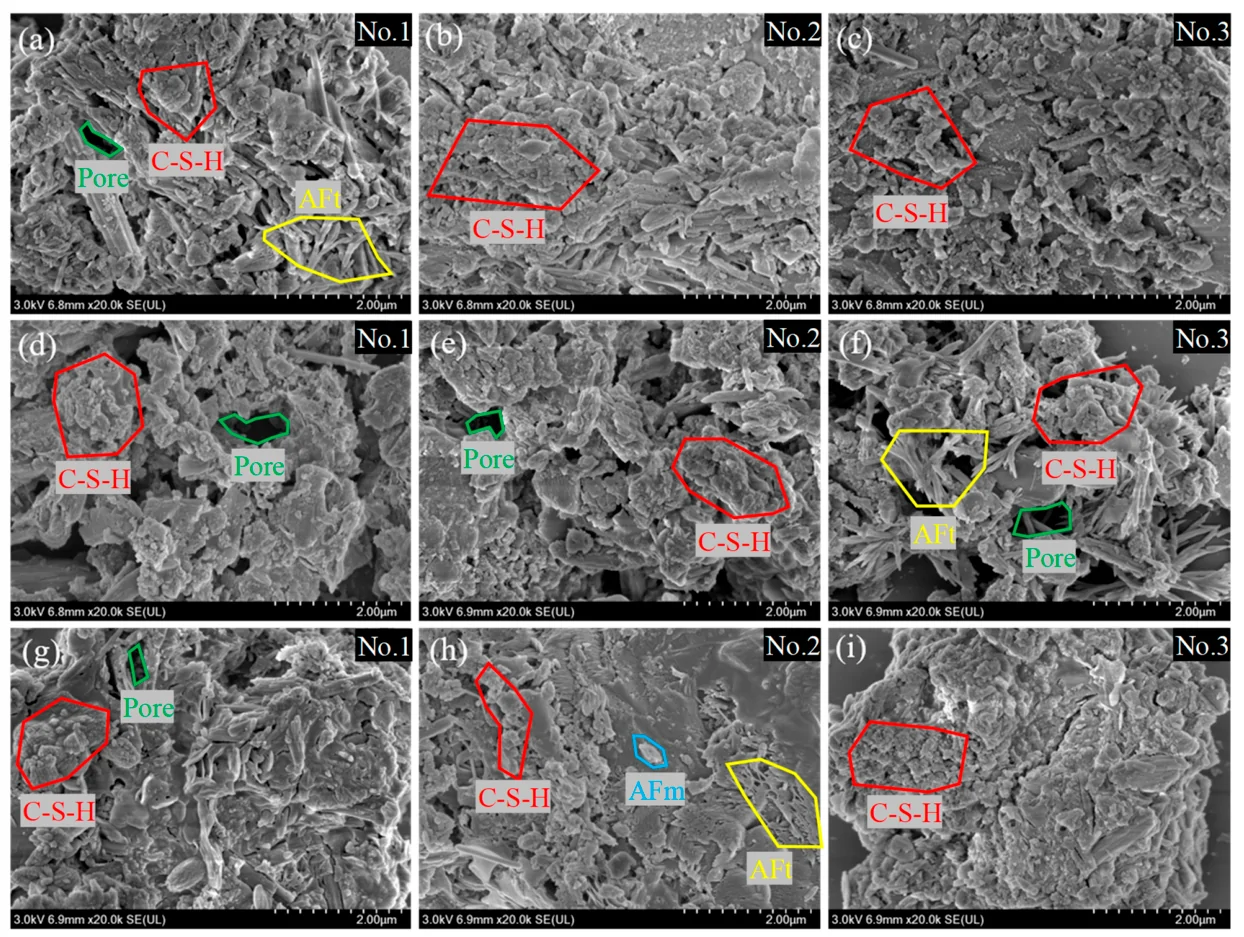
(**a**–**c**) SEM images of different samples after hydration for 1 d, (**d**–**f**) SEM images of different samples after hydration for 10 d, and (**g**–**i**) SEM images of different samples after hydration for 28 d.

SEM images collected at 1 d, 10 d and 28 d record the morphological changes in hydration products at different curing ages (Figure 5). It can be seen that the early AFt crystal skeleton is gradually filled by continuously generated C-S-H gel [37]. The morphological differences in samples with different mixing ratios reflect the regulating effect of amide on hydration product crystallization and matrix compactness (Figure 5a,b). Consistent with the XRD test results, obvious morphological changes related to AFt-to-AFm transformation can be observed in groups with low aluminum sulfate content [38]. Among all tested samples, No. 3 shows better overall micromorphology at 28 d, with fewer adverse phase transformation characteristics, which indicates that the matched addition of aluminum sulfate and amide helps maintain a relatively intact microstructure, consistent with its highest residual compressive strength among all modified mixtures (Figure 5c). These observations are consistent with the hypothesis that the selected aluminum sulfate to amide ratio contributes to a relatively well preserved microstructure, although SEM only captures localized regions and cannot be considered statistically representative of the bulk specimen [39].

### 3.5. Thermal Analysis of Hydration Products

**Figure 6:**
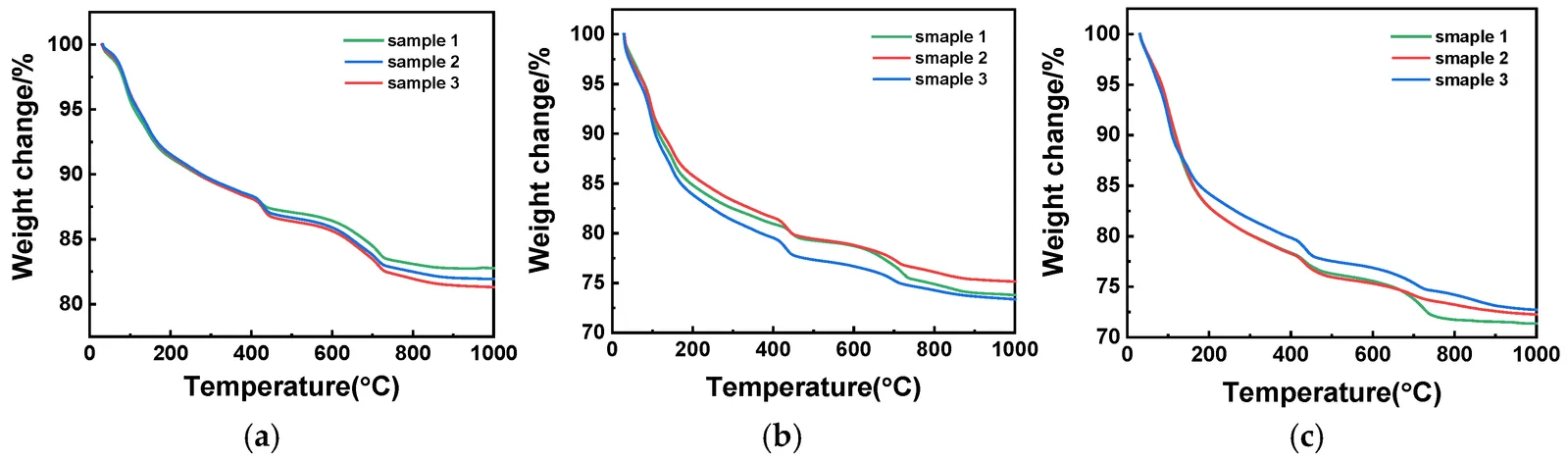
Thermogravimetric analysis of different samples (**a**) in 1 d, (**b**) in 10 d and (**c**) in 28 d.

Thermal analysis provides crucial insights into the decomposition characteristics and thermal stability of hydration products in cement pastes modified with aluminum sulfate (AS) and amide compound. Thermogravimetric (TGA) analysis was performed on a Netzsch STA 449 F3 instrument under N_2_ atmosphere from 30 to 900 °C at a heating rate of 10 °C/min. The TGA curves (Figure 6) reveal distinct mass loss stages corresponding to the decomposition of specific hydration phases. The initial mass loss observed between 100 °C and 200 °C is attributed to the dehydration of ettringite (AFt) and C-S-H gel, while the significant mass loss between 400 °C and 550 °C corresponds to the dehydroxylation of portlandite (Ca(OH)_2_). It should be noted that the samples analyzed at 10 d were extracted from the same batch specimens prepared for the 10 d mechanical tests; this interval allowed for sufficient sample drying and stabilization prior to analysis without significantly altering the mature phase assemblage relevant to early-age performance.

At the early hydration stage (1 d), Sample 3 (moderate amide/high AS) exhibits the most pronounced mass loss in the low-temperature region (100–200 °C), indicating substantial formation of AFt and C-S-H gel. This observation aligns with its superior 1-day compressive strength (17.05 MPa) and confirms the effective synergistic action between AS and the amide compound in promoting early hydration product precipitation. Quantitative mass loss data in Table 3 further confirm this trend: Sample 3 exhibits the highest Stage I loss (1.8%) at 1 d, followed by Sample 2 (1.4%) and Sample 1 (1.2%), consistent with its superior 1-day compressive strength. The magnitude of this mass loss suggests that the optimized dosage ratio facilitates the development of a well-crystallized ettringite framework, which contributes significantly to early strength development.

As hydration progresses to 10 d, the TGA curves demonstrate continued hydration product accumulation. Sample 3 maintains the highest total mass loss in the AFt decomposition region, consistent with its remarkable 10 d compressive strength (27.37 MPa). The sustained high AFt content, coupled with ongoing C-S-H formation, supports the development of a dense microstructure that yields excellent mechanical performance. In contrast, Sample 2 (high amide/low AS) shows reduced mass loss in this region, reflecting insufficient AFt formation due to limited aluminum sulfate availability, which correlates with its lower compressive strength at this stage. As listed in Table 3, Sample 3 maintains the largest Stage I mass loss (2.5%) at 10 d, while Sample 2 and Sample 1 show 2.1% and 1.7%, respectively, supporting the proposed microstructural densification.

At 28 d of hydration, the thermal analysis reveals important insights into phase stability. All samples containing additives show evidence of AFt to AFm conversion, reflected by the evolving profile of the low-temperature mass loss step. However, Sample 3 retains a higher absolute content of AFt relative to other formulations, contributing to its highest absolute 28 d strength. Nevertheless, the relative strength loss from 10 to 28 d is most pronounced in Sample 3, reflecting that even a relatively better preserved AFt phase cannot fully suppress the macroscopic effects of ongoing AFt to AFm conversion over extended hydration. Regarding the portlandite decomposition step (around 500 °C), Sample 3 exhibits a moderately intense Ca(OH)_2_ decomposition signal, indicating balanced silicate hydration where sufficient Ca(OH)_2_ is generated without excessive accumulation. This balanced reaction contributes to the formation of a stable and durable microstructure that maintains good long-term performance.

The thermal analysis results strongly support the proposed mechanism where the amide compound functions as a molecular-scale regulator that fine-tunes the crystallization process. The optimal dosage combination in Sample 3 promotes the formation of well-crystallized, thermally stable hydration products, while preventing the formation of metastable phases that would undergo detrimental transformations during prolonged hydration.

### 3.6. Reaction Processes and Mechanisms in the Cement-Aluminum Sulfate-Amide Compound System

**Figure 7:**
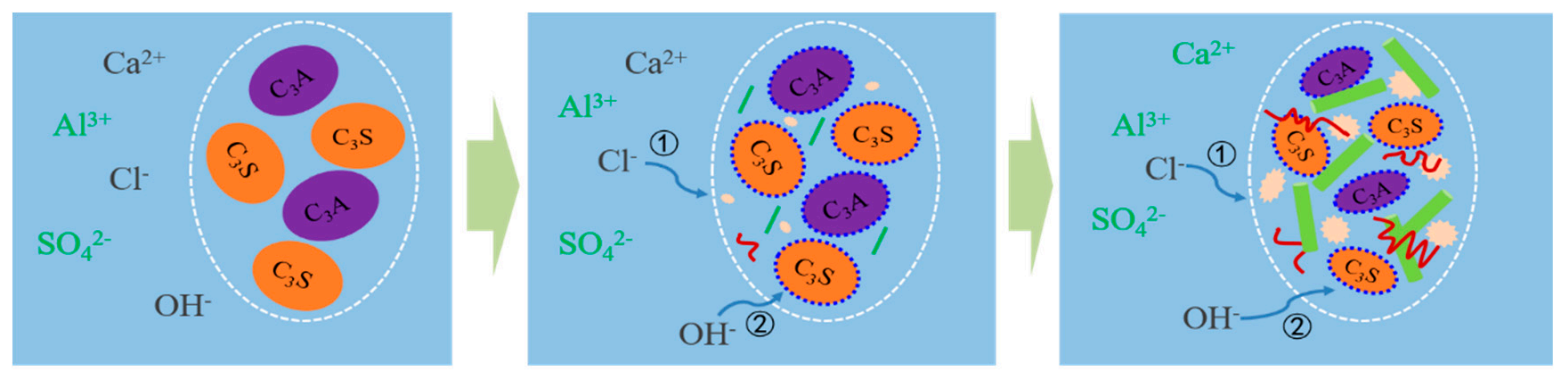
Reaction mechanisms in the cement-aluminum sulfate-amide compound system.

The reactions within this system are multi-stage, multi-phase processes involving ionic dissolution, precipitation, adsorption, and transformation (Figure 7). The following equations detail the core reaction mechanisms [40,41,42].

(1) Dissolution of aluminum sulfate and early ettringite formation (ionic driving force)

Aluminum sulfate first dissolves rapidly, providing crucial aluminum and sulfate ions to the system:

Al_2_(SO_4_)_3_·18H_2_O → 2Al^3+^ + 3SO_4_^2−^ + 18H_2_O

The dissolved Al^3+^ immediately reacts with Ca^2+^ and OH^−^ ions from the hydration of C_3_S in the cement clinker, and SO_4_^2−^ from dissolved gypsum (CaSO_4_·2H_2_O) to form AFt:

2Al^3+^ + 6OH^−^ + 3Ca^2+^ + 3SO_4_^2−^ + 26H_2_O → 3CaO·Al_2_O_3_·3CaSO_4_·32H_2_O (AFt)

(2) Adsorption and complexation by the amide compound (interface regulation)

Surface adsorption: The amide group (represented as R−CONH_2_) adsorbs onto cement mineral surfaces (e.g., C_3_A, C_3_S).

R-CONH_2_ + ≡Surface → ≡Surface⋯HN−CO−R

(where ≡Surface represents the cement mineral surface).

Calcium ion complexation: R-CONH_2_ with Ca^2+^ in the pore solution, forming soluble complexes.

R−CONH_2_ + Ca^2+^ ⇌ [Ca(R−CONH_2_)]^2+^

This complex can act as a heterogeneous nucleation site for hydration products like C-S-H gel, promoting their precipitation.

(3) Coordinated C–S–H precipitation

Under the influence of the additives, the primary strength-giving C-S-H gel forms through the hydration of C_3_S and C_2_S:

2(3CaO·SiO_2_) + 6H_2_O → 3CaO·2SiO_2_·3H_2_O + 3Ca(OH)_2_

(abbreviated as: 2C_3_S + 6H → C-S-H + 3CH)

(4) Controlled AFt-to-AFm Conversion

During prolonged hydration, as the available sulfate ions are depleted, the metastable AFt reacts with remaining unhydrated tricalcium aluminate (C_3_A) to form stable monosulfoaluminate (AFm).

3CaO·Al_2_O_3_·3CaSO_4_·32H_2_O + 2(3CaO·Al_2_O_3_) → 3(3CaO·Al_2_O_3_·CaSO_4_·12H_2_O)

(abbreviated as: 2C_3_A + C_6_AS_3_H_32_ + 4H → 3C_4_ASH_12_)

This phase conversion is accompanied by a significant reduction in solid volume and an increase in total porosity, leading to increased micro-porosity and a macroscopic strength decrease.

**Figure 8:**
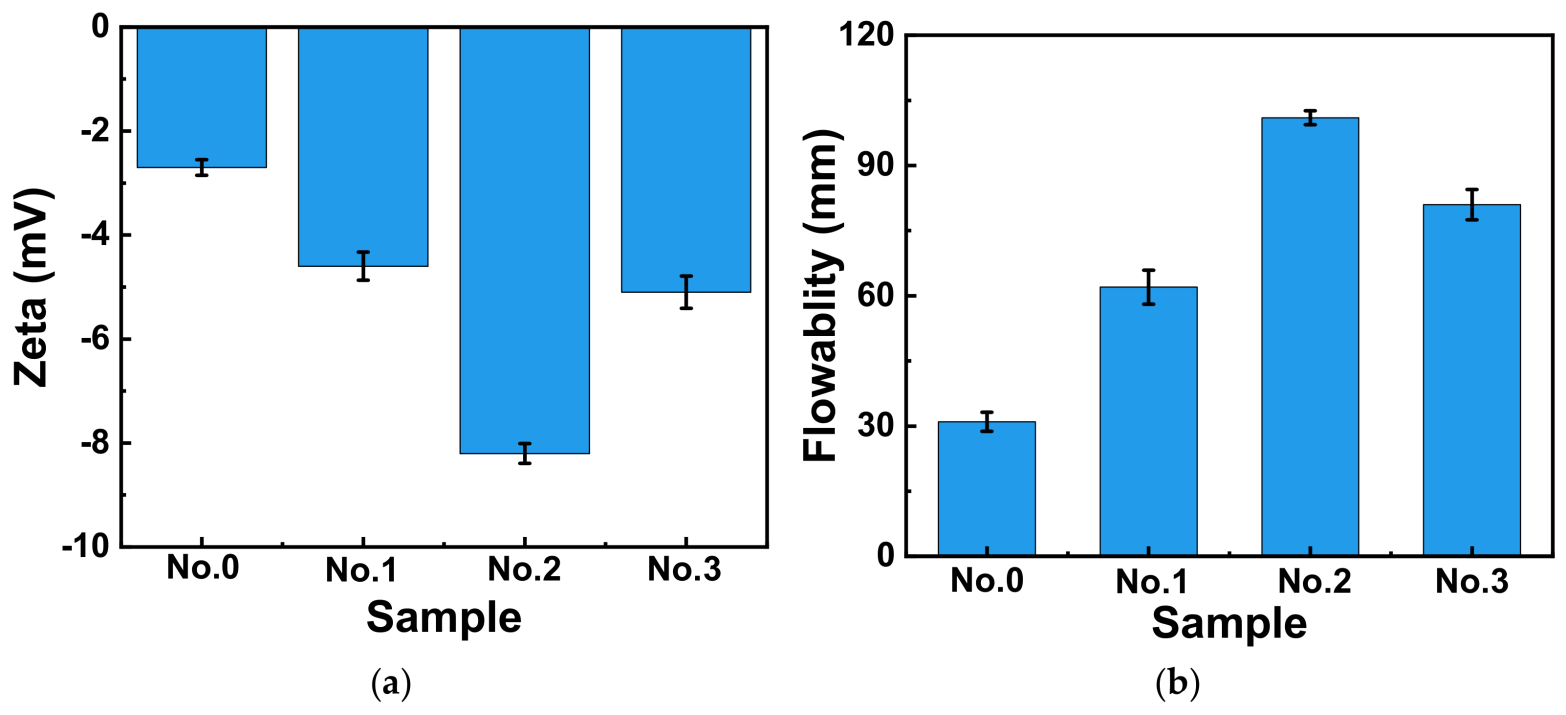
(**a**) Zeta potential and (**b**) flowability of cement pastes with different aluminum sulfate–amide dosages, indicating the interfacial electrostatic regulation induced by amide adsorption under varying AS/amide ratios.

Zeta potential measurements are consistent with and supportive of the proposed interfacial regulation role of the amide compound (Figure 8a). Compared with the blank sample, all AS–amide modified systems exhibit a more negative zeta potential, indicating pronounced adsorption of organic species on cement particle surfaces. Notably, the high-amide/low-AS system (No. 2) shows the most negative zeta potential, suggesting excessive surface coverage and strong electrostatic repulsion, which is detrimental to the formation of a well-interlocked hydration skeleton. In contrast, the optimized formulation (No. 3) presents a moderately shifted zeta potential, reflecting a balanced interfacial modification that promotes nucleation while avoiding over-stabilization of the particle dispersion. This result provides direct experimental evidence supporting the proposed molecular-scale regulatory mechanism.

Figure 8b illustrates the flowability of cement pastes as influenced by different aluminum sulfate (AS) to amide compound dosage ratios, providing a direct correlation with the zeta potential data. The results show that Sample No. 2 (high amide/low AS) exhibits the highest flowability, which aligns with its most negative zeta potential, indicating excessive amide adsorption causes strong electrostatic repulsion and over-dispersion of cement particles. In contrast, Sample No. 1 (high AS/high amide) demonstrates relatively lower flowability, as the high amide content, combined with rapid ion precipitation from AS, adversely affects rheology. Sample No. 3 (high AS/moderate amide) achieves an intermediate and optimal flowability, where the balanced AS/amide ratio promotes effective interfacial modification without disrupting the formation of a cohesive early-age microstructure.

In order to elucidate the interaction mechanisms between the amide compound and cement mineral phases at the atomic and electronic scale, density functional theory (DFT) calculations were performed. All simulations were performed using CASTEP with the GGA-PBE exchange-correlation functional and ultrasoft pseudopotentials. Surface models were constructed from bulk C_3_A (110) and C_3_S (001) facets—the dominant exposed surfaces during early hydration—with a 15 Å vacuum layer to prevent periodic interactions. The Monkhorst-Pack k-point grid was set to 2 × 2 × 1 for geometry optimization and 4 × 4 × 1 for density of states calculations, with a plane-wave cutoff energy of 450 eV. Convergence thresholds were 1 × 10^−5^ eV/atom for energy, 0.03 eV/Å for maximum force, and 0.05 GPa for maximum stress. Adsorption energy was calculated as *E*_ads_ = *E*_total_ − (*E*_slab_ + *E*_molecule_), where *E*_total_ is the energy of the adsorbed system, *E*_slab_ the clean surface energy, and *E*_molecule_ the isolated molecule energy [43,44].

**Figure 9:**
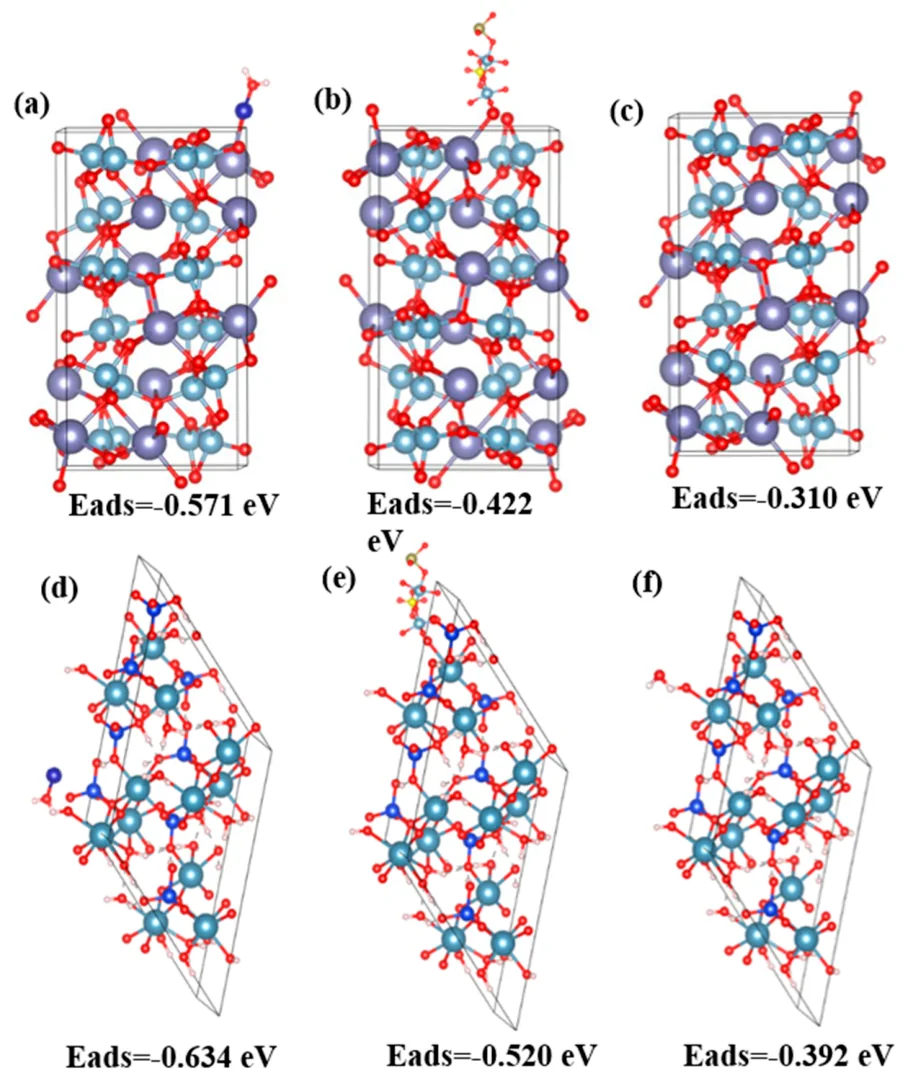
DFT simulation for the structure and the adsorption of (**a**,**d**) acetamide, (**b**,**e**) aluminum sulfate, (**c**,**f**) H_2_O onto C_3_A and C_3_S. Atomic color code: Ca = purple, Al/Si = light blue, O = red, N = dark blue, H = white.

Regarding the model molecule: acetamide was selected as a computationally tractable analog of the tertiary amide moieties (-CON<) in our actual copolymer. Both share the identical electron-donating carbonyl oxygen and nitrogen coordination sites responsible for Ca^2+^ complexation and surface adsorption; the methyl groups in acetamide reasonably mimic the local electronic environment of the copolymer’s amide linkages without the prohibitive computational cost of polymer chain modeling. The results, summarized in Figure 9, provide fundamental insights into the adsorption behaviors that underpin the macroscopic phenomena observed in this study. Figure 9 presents a comparative analysis of the optimized adsorption configurations and electronic structures of acetamide (as a model amide compound), an aluminum sulfate ion cluster, and a water molecule on the dominant surfaces of tricalcium aluminate (C_3_A) and tricalcium silicate (C_3_S).

The DFT simulations reveal distinct adsorption configurations for the different molecules. As shown in Figure 9a,d, the acetamide molecule adsorbs stably on both C_3_A and C_3_S surfaces primarily through its polar amide group (-CONH_2_). The oxygen and nitrogen atoms of the amide group form coordinated bonds with the calcium atoms on the mineral surfaces, indicating a specific chemical interaction rather than a mere physical attachment. In contrast, the adsorption of the aluminum sulfate ion cluster (Figure 9b,e) involves ionic interactions with the surface, while the water molecule (Figure 9c,f) exhibits a much weaker and simpler adsorption mode, primarily through hydrogen bonding and dipole interactions.

A critical finding from these calculations is the quantitative hierarchy of adsorption energies, which follows the order: acetamide > aluminum sulfate cluster > water molecule. This order has profound implications for the proposed mechanism. The superior adsorption energy of acetamide confirms its strong affinity for cement mineral surfaces, providing a theoretical foundation for its role as a molecular-scale regulator. This strong adsorption suggests that acetamide molecules can effectively compete for and occupy active sites on the mineral surfaces, potentially displacing pre-adsorbed water molecules. This process disrupts the diffuse double-layer structure at the solid–liquid interface, promoting particle flocculation and altering the nucleation environment for hydration products like ettringite and C-S-H gel. The stronger adsorption of acetamide compared to the aluminum sulfate ions further implies that the amide compound plays a dominant role in modifying the interface, thereby fine-tuning the crystallization kinetics driven by the ionic precipitation from aluminum sulfate.

### 3.7. Limitations

It should be emphasized that this study only incorporates three discrete AS-amide blended formulations alongside a blank control, without continuous intermediate dosage gradients. The superior overall performance of Sample No. 3 merely illustrates a performance trend within the present limited experimental matrix and cannot identify the globally optimal matching ratio between aluminum sulfate and amide. Full-factorial gradient experiments incorporating additional intermediate mixing proportions are therefore required to establish quantitative dosage-performance correlations and precisely determine the optimal dosage window. While multi-scale characterizations robustly validate the qualitative synergistic molecular regulatory mechanism-including preferential amide adsorption, AFt stabilization by aluminum sulfate, and suppression of AFt to AFm transformation-the quantitative thresholds for amide-induced retardation and the stabilizing effect of aluminum sulfate remain unclarified, which necessitates expanded testing series in subsequent investigations.

It should also be noted that all accelerator-modified samples experience obvious strength retrogression from 10 d to 28 d caused by AFt-to-AFm conversion. Although the optimized high-AS moderate-amide formulation (Sample No. 3) retains the maximum 28 d residual strength and meets standard shotcrete strength requirements, its 36.5% relative strength loss still restricts its service performance for permanent structural components. Subsequent multi-gradient dosage optimization will focus on inhibiting AFt decomposition to further mitigate long-term strength degradation.

## 4. Conclusions

This work elucidates the synergistic mechanism between aluminum sulfate and an amide compound in controlling cement hydration and microstructure development. The results establish that the dosage ratio of these components critically determines the setting behavior, strength development, and long-term performance of the cementitious system. Among the dosage combinations tested in this work, the mixture with high aluminum sulfate and moderate amide content accelerates ettringite precipitation and uniform C-S-H gel formation, achieving the highest absolute early and 28 d strengths among the modified systems, although the relative strength retrogression from 10 to 28 d remains the largest within this series. The amide compound functions as a molecular regulator through specific adsorption onto cement mineral surfaces and complexation with calcium ions, thereby fine-tuning the nucleation kinetics of hydration products. The combined characterization results are consistent with comparatively improved stability of the hydration phase assemblage and a delayed tendency for ettringite to AFm conversion in the most favorable formulation identified within this study. Theoretical calculations provide fundamental support for the proposed mechanism, revealing the strong adsorption affinity of the amide compound compared to aluminum sulfate and water. Collectively, these findings provide a coherent and mechanistically plausible framework for understanding the coordinated interaction between ionic precipitation and surface-mediated nucleation, which can inform the further development of alkali-free accelerators with improved performance for cement-based materials.

## Figures and Tables

**Table 1:** Mix proportions of cement pastes (amounts per 400 g of cement).

Number	Water (g)	Additive (7%)					
		Aluminum Sulfate (g)	high Alumina Salt (g)	Amidation Reaction Product (g)	Water	Total Effective Water (g)	Effective w/c Ratio
No. 0 sample	140	/	/	/	/	140	0.35
No. 1 sample	130.76	14	1.96	2.8	9.24	140	0.35
No. 2 sample	125.16	8.4	1.96	2.8	14.84	140	0.35
No. 3 sample	129.36	14	1.96	1.4	10.64	140	0.35

**Table 2:** The compressive strength results.

Sample	Compressive Strength (MPa)								
	1 d	SD	CV%	10 d	SD	CV%	28 d	SD	CV%
No. 0	6.41	0.53	5.93	7.66	0.42	5.87	8.53	0.63	5.86
No. 1	9.37	0.71	5.98	19.81	0.36	6.01	16.50	0.43	6.00
No. 2	11.41	0.45	5.96	20.87	0.75	5.99	15.26	0.52	6.03
No. 3	17.05	0.81	5.98	27.37	0.52	5.99	17.38	0.61	5.98

Note: Relative compressive strength loss from 10 d to 28 d: No. 1 = 16.7%, No. 2 = 26.9%, No. 3 = 36.5%.

**Table 3:** The ratio of quality loss at each stage.

Age	Sample	Stage I	Stage II	Stage III
1 d	Sample 1	1.2%	3.5%	5.5%
1 d	Sample 2	1.4%	3.8%	5.4%
1 d	Sample 3	1.8%	3.9%	4.8%
10 d	Sample 1	1.7%	4.1%	8.5%
10 d	Sample 2	2.1%	4.9%	9.0%
10 d	Sample 3	2.5%	5.4%	8.8%
28 d	Sample 1	2.2%	4.9%	8.5%
28 d	Sample 2	2.6%	5.7%	8.3%
28 d	Sample 3	2.9%	6.1%	7.7%

## Data Availability

The original contributions presented in this study are included in the article. Further inquiries can be directed at the corresponding authors.
